# Interventions Targeting Glucocorticoid-Krüppel-like Factor 15-Branched-Chain Amino Acid Signaling Improve Disease Phenotypes in Spinal Muscular Atrophy Mice

**DOI:** 10.1016/j.ebiom.2018.04.024

**Published:** 2018-05-04

**Authors:** Lisa M. Walter, Marc-Olivier Deguise, Katharina E. Meijboom, Corinne A. Betts, Nina Ahlskog, Tirsa L.E. van Westering, Gareth Hazell, Emily McFall, Anna Kordala, Suzan M. Hammond, Frank Abendroth, Lyndsay M. Murray, Hannah K. Shorrock, Domenick A. Prosdocimo, Saptarsi M. Haldar, Mukesh K. Jain, Thomas H. Gillingwater, Peter Claus, Rashmi Kothary, Matthew J.A. Wood, Melissa Bowerman

**Affiliations:** a Institute of Neuroanatomy and Cell Biology, Hannover Medical School, Hannover, Germany; b Center of Systems Neuroscience, Hannover, Germany; c Ottawa Hospital Research Institute, Regenerative Medicine Program, Ottawa, ON, Canada; d Department of Medicine and Cellular and Molecular Medicine, University of Ottawa, Ottawa, ON, Canada; e Department of Physiology, Anatomy and Genetics, University of Oxford, Oxford, United Kingdom; f Medical Research Council, Laboratory of Molecular Biology, Cambridge, United Kingdom; g Euan MacDonald Centre for Motor Neurone Disease Research, University of Edinburgh, Edinburgh, United Kingdom; h Centre for Integrative Physiology, University of Edinburgh, Edinburgh, United Kingdom; i Case Cardiovascular Research Institute, Case Western Reserve University School of Medicine, University Hospitals Case Medical Center, Cleveland, OH, USA; j Gladstone Institute of Cardiovascular Disease, San Francisco, CA, USA; k Department of Medicine, Division of Cardiology University of California, San Francisco, CA, USA

**Keywords:** Spinal muscular atrophy, KLF15, Glucocorticoids, Branched-chain amino acids, Metabolism, Therapy

## Abstract

The circadian glucocorticoid-Krüppel-like factor 15-branched-chain amino acid (GC-KLF15-BCAA) signaling pathway is a key regulatory axis in muscle, whose imbalance has wide-reaching effects on metabolic homeostasis. Spinal muscular atrophy (SMA) is a neuromuscular disorder also characterized by intrinsic muscle pathologies, metabolic abnormalities and disrupted sleep patterns, which can influence or be influenced by circadian regulatory networks that control behavioral and metabolic rhythms. We therefore set out to investigate the contribution of the GC-KLF15-BCAA pathway in SMA pathophysiology of Taiwanese *Smn*^*−/−*^*;SMN2* and *Smn*^*2B/−*^ mouse models. We thus uncover substantial dysregulation of GC-KLF15-BCAA diurnal rhythmicity in serum, skeletal muscle and metabolic tissues of SMA mice. Importantly, modulating the components of the GC-KLF15-BCAA pathway via pharmacological (prednisolone), genetic (muscle-specific Klf15 overexpression) and dietary (BCAA supplementation) interventions significantly improves disease phenotypes in SMA mice. Our study highlights the GC-KLF15-BCAA pathway as a contributor to SMA pathogenesis and provides several treatment avenues to alleviate peripheral manifestations of the disease. The therapeutic potential of targeting metabolic perturbations by diet and commercially available drugs could have a broader implementation across other neuromuscular and metabolic disorders characterized by altered GC-KLF15-BCAA signaling.

## Highlights

• SMA is a neuromuscular disease characterized by motoneuron loss, muscle abnormalities and metabolic perturbations.
• The regulatory GC-KLF15-BCAA pathway is dysregulated in serum and skeletal muscle of SMA mice during disease progression.
• Modulating GC-KLF15-BCAA signaling by pharmacological, dietary and genetic interventions improves phenotype of SMA mice.

Spinal muscular atrophy (SMA) is a devastating and debilitating childhood genetic disease. Although nerve cells are mainly affected, muscle is also severely impacted. The normal communication between the glucocorticoid (GC) hormone, the protein KLF15 and the dietary branched-chain amino acids (BCAAs) maintains muscle and whole-body health. In this study, we identified an abnormal activity of GC-KLF15- BCAA in blood and muscle of SMA mice. Importantly, targeting GC-KLF15-BCAA activity with an existing drug or a specific diet improved disease progression in SMA mice. Our research uncovers GCs, KLF15 and BCAAs as therapeutic targets to ameliorate SMA muscle and whole-body health.

## 1 Introduction

Transcriptional regulation is one of the main control mechanisms of metabolic processes [1]. Krüppel-like factor 15 (KLF15) is a transcription factor expressed in a multitude of metabolic tissues including skeletal muscle [2] where it is involved in regulation of lipid [3], glucose [4], and amino acid metabolism [5]. Specifically, *KLF15* displays a diurnal pattern of expression, and regulates branched-chain amino acids (BCAA) metabolism and utilization in a circadian fashion [5]. BCAAs (isoleucine, leucine and valine) are a major source of essential amino acids in muscle (35%) [6]. Accumulating evidence in various species suggest that BCAAs promote survival, longevity [7,8] and repair of exercise- and sarcopenia-induced muscle damage [9,10]. Both KLF15 and BCAAs are modulated by circadian secretion of glucocorticoids (GCs) and activity of the glucocorticoid receptor (GR) [11,12]. GCs are also used surreptitiously by endurance athletes for their ergogenic properties [13] and as treatment for genetic muscle pathologies [14].

The neuromuscular disease spinal muscular atrophy (SMA) is the most common autosomal recessive disorder leading to infant mortality [15]. It is characterized by degeneration of α-motoneurons in the ventral horn of the spinal cord as well as progressive muscle weakness and atrophy [16,17]. SMA is a monogenic disease caused by homozygous deletions or mutations within the *survival motor neuron 1* (*SMN1*) gene [18,19]. Complete loss of SMN is embryonic lethal in mice [20]. However, humans have at least one copy of the highly homologous *SMN2* gene, which generates a low amount of functional protein that allows for embryonic development, while not being sufficient for complete rescue in the event of *SMN1* loss. This is due to a nucleotide transition in *SMN2* that favors alternative splicing of exon 7 and production of a non-functional truncated protein [19,21,22]. Whilst several cellular functions for SMN have been defined [23, 24, 25], it remains elusive why a lack of the ubiquitously expressed SMN results in the canonical SMA phenotype.

Although motoneurons are the primary cellular targets in SMA, a number of tissues outside the central nervous system (CNS) also contribute to disease pathophysiology [26], with skeletal muscle being the most prominently afflicted [27]. As muscle plays an important role in maintaining systemic energy homeostasis [28], intrinsic muscle defects can have severe consequences on whole-body metabolism. Various studies in SMA animal models and patients report metabolic abnormalities such as abnormal fatty acid metabolism [29, 30, 31], defects in glucose metabolism and pancreatic development [32,33] and the coexistence of diabetes mellitus and diabetic ketoacidosis in SMA patients [34,35]. The observation that dietary supplementation improves lifespan of SMA mice [36, 37, 38] further supports the hypothesis that metabolic perturbations contribute to SMA pathology. We thus postulate that intrinsic metabolic defects in skeletal muscle play a contributory role in whole-body metabolic perturbations in SMA.

Here, we identify dysregulation of the GC-KLF15-BCAA pathway in skeletal muscle as a key pathological event in SMA. Notably, we demonstrate that pharmacological and dietary interventions that modulate this pathway lead to significant phenotypic improvements in SMA mice. Our results reveal the importance of the GC-KLF15-BCAA axis in SMA pathogenesis and highlight its potential as a therapeutic target to attenuate muscle and metabolic disturbances in SMA. The accessibility and ease of administration of the dietary and drug treatments identified in our study make them exciting clinical avenues to investigate not only in SMA patients but also in individuals with other neuromuscular and neurodegenerative diseases where GC-KLF15-BCAA signaling may be altered.

## 2 Materials and Methods

### 2.1 Animals

The Taiwanese *Smn*^*−/−*^*;SMN2* (FVB/N background, FVB·Cg-Smn1tm1HungTg(SMN2)2Hung/J, RRID: J:59313), *Smn*^*2B/−*^ (C57BL/6 background, RRID: not available) and *KLF15 MTg* (C57BL/6 background, RRID: not available) mice were housed either in individual ventilated cages in the typical holding rooms of the animal facility or in circadian isolation cages (12 h light:12 h dark cycle, LD12:12). Experiments with the *Smn*^*−/−*^*;SMN2* and *Klf15 MTg* mice were carried out in the Biomedical Sciences Unit, University of Oxford, according to procedures authorized by the UK Home Office (Animal Scientific Procedures Act 1986). Experiments with the *Smn*^*2B/−*^ mice were carried out at the University of Ottawa Animal Facility according to procedures authorized by the Canadian Council on Animal Care. Prednisolone (5 mg tablets, Almus) was dissolved in water (1 tablet in 5 mL) and administered by gavage on every second day starting at P0 until death in the severe Taiwanese *Smn*^*−/−*^*;SMN2* SMA mouse model. In the *Smn*^*2B/−*^ mouse model, prednisolone or saline was administered by gavage every two days from P0 to P20. For the *Smn*^*2B/−*^ mouse model treatment, weaned mice were given daily wet chow at the bottom of the cage to ensure proper access to food. BCAA peptides (Myprotein) were diluted in water (300 mg in 2 mL) and administered to the severe Taiwanese *Smn*^*−/−*^*;SMN2* SMA mice by gavage starting at P5. Pip6a-PMO and Pip6a-scrambled compounds were delivered by facial vein injections at P0 and P2 (10 μg/g diluted in 0.9% saline) to WT and severe Taiwanese *Smn*^*−/−*^*;SMN2* SMA mice. Prednisolone and BCAAs were administered to the animals around the same time each day. Litters were randomly assigned to treatment prior to birth. For survival studies, animals were weighed daily and culled upon reaching their defined humane endpoint. To reduce total number of animals used, the fast-twitch *tibialis anterior* and *triceps* muscles from the same animal were used interchangeably for respective molecular and histological analyses. Sample sizes were determined based on similar studies with SMA mice.

### 2.2 Peptide-PMO Synthesis

Pip6a Ac-(RXRRBRRXRYQFLIRXRBRXRB)-COOH was synthesized and conjugated to a PMO chemistry as previously described [39]. The full length *SMN2* enhancing PMO (5′-ATTCACTTTCATAATGCTGG-3′) and scrambled PMO (5′- TACGTTATATCTCGTGATAC-3′) sequences were purchased from Gene Tools LLC.

### 2.3 qPCR

Skeletal muscles were harvested at several time-points during disease progression and immediately flash frozen. For circadian experiments, liver, heart, white and brown adipose tissue (WAT and BAT), spinal cord and *tibialis anterior* muscles were harvested from P2 and P7 pups every 4 h over a 24 h period (ZT1 = 9 am, ZT5 = 1 pm, ZT9 = 5 pm, ZT13 = 9 pm, ZT17 = 1 am, ZT21 = 5 am). RNA was extracted with the RNeasy MiniKit (Qiagen) except for WAT and BAT where the RNeasy Lipid Tissue MiniKit (Qiagen) was used. Reverse transcription was performed using the High-Capacity cDNA Reverse Transcription Kit (ThermoFisher Scientific). qPCR was performed either using TaqMan Gene Expression Mastermix (ThermoFisher Scientific) or SYBR green Mastermix (ThermoFisher Scientific) and primers were from Integrated DNA Technologies (see Supplementary Experimental Procedures). For SYBR green qPCRs, *RNA polymerase II polypeptide J* (*PolJ*), was used as a validated housekeeping gene. *PolJ* has previously been demonstrated as being stably expressed between tissues and in different pharmacological conditions [40]. For circadian experiments and TaqMan qPCRs, housekeeping genes for each tissue were determined using the Mouse geNorm Kit and qbase+ software (Primerdesign).

### 2.4 PCR Arrays

RNA from skeletal muscle was extracted using the RNeasy Microarray Tissue Mini Kit (Qiagen). cDNA was made using RT^2^ First Strand Kit (Qiagen). qPCRs were performed using Mouse Amino Acid Metabolism I & II PCR arrays (PAMM-129Z and PAMM-130Z, SABiosciences). Data was analyzed with the RT Profiler PCR Array Data Analysis version 3.5 and mRNA expression was normalized to the geometric average of the two most stably expressed housekeeping genes between all samples.

### 2.5 Immunoblots With Mouse Tissues

Triceps were isolated from P7 *Smn*^*−/−*^*;SMN2* mice and healthy control littermates and snap frozen in liquid nitrogen. The tissue was lysed in 200 μL RIPA buffer (150 mM NaCl, 50 mM Tris, 0.5% sodium deoxycholate, 0.1% TX-100, 5 mM sodium pyrophosphate, 2 mM β-glycerophosphate, 1 × EDTA-free protease inhibitor (Roche), 1 × PhosSTOP phosphatase inhibitor (Roche), pH 7.5) using Precellys 24 homogenizer (Stretton Scientific). Total protein (20 μg per lane) was resolved on Tris-glycine SDS-PAGE and transferred to PVDF membrane. The following antibodies were used: p70 S6 kinase (#2708, RRID: AB_390722), S6 Ribosomal Protein (#2217, RRID: AB_331355), Phospho-S6 Ribosomal Protein (Ser235/236, #2211, RRID: AB_331679) (all 1:1000, Cell Signaling Technology), and goat anti-rabbit IRDye 800CW (#827–08365, LI-COR Biosciences, RRID: AB_10796098). The membranes were imaged and quantified using ImageStudio and LI-COR Odyssey Fc (LI-COR Biosciences). Band intensities were normalized to total protein as determined by Fast Green (FG) stain (125 μM Fast Green FCF, 6.7% acetic acid, 30% methanol) [41]. Each biological sample (n) was run in 3–4 technical replicates and the average of all technical replicates was used to determine the final relative expression for each biological sample.

### 2.6 Corticosterone ELISA

Analysis of corticosterone content in serum was performed with an ELISA kit (#ab108821, Abcam) following the manufacturer's instructions. Serum samples were diluted 1:10.

### 2.7 BCAA Content in Muscle and Serum

Levels of valine, leucine and isoleucine were measured in muscle and serum by high-performance liquid chromatography (HPLC) (AltaBiosciences, Birmingham, UK). Skeletal muscles were pooled to reach a minimum weight of 100 mg and sera were pooled to reach a minimum volume of 150 μL.

### 2.8 Neuromuscular Junction (NMJ) Immunohistochemistry

NMJs were stained as previously described [42]. Briefly, whole *tibialis anterior* muscle was harvested and fixed in 4% paraformaldehyde (PFA) for 15 min. Muscles were incubated with α-bungarotoxin (α-BTX) conjugated to tetramethylrhodamine (#BT00012, Biotium, 1:100, RRID: not available) at RT for 30 min with ensuing PBS washes. Muscles were incubated in blocking solution (0.2% Triton-X, 2% BSA, 0.1% sodium azide) at RT for 1 h. Nerve terminals were stained with antibodies against synaptic vesicle 2 (#SV2 (Supernatant 1 mL), Developmental Studies Hybridoma Bank, 1:100, RRID: AB_2315387) and neurofilament NF-M (#2H3, Supernatant 1 mL), Developmental Studies Hybridoma Bank, 1:100, RRID: AB_2314897) overnight at 4 °C. Following three PBS washing steps, the tissue was incubated with an Alexα Fluor 488 goat anti-mouse antibody (#A-21141, Molecular Probes, 1:500, RRID:AB_141626) at RT for 1 h. Finally, 2–3 thin filets per muscle were sliced and mounted in Fluoromount-G (Southern Biotech). Images were taken with a confocal microscope, with a 20× objective, equipped with filters suitable for FITC/Cy3 fluorescence. The experimenter quantifying NMJ morphology and innervation was blinded to the genotype of the animals until all measurements were finalized.

### 2.9 Human Samples

Skeletal muscle biopsies from SMA patients and controls were obtained from two different biobanks (Fondazione IRCCS Istituto Neurologico “C. Besta” and Fondazione Ospedale Maggiore Policlinico Mangiagalli en Regina Elena, IRCCS) [43]. As previously described, protein was extracted in RIPA buffer with 10% protease inhibitor cocktail (Sigma). Equal amounts of total protein were loaded and blocked in Odyssey buffer (LI-COR Biosciences). Membranes were incubated overnight with the primary antibody goat anti-KLF15 (#ab2647, Abcam, 1:1000, RRID: AB_303232). The secondary antibody was rabbit anti-Goat IgG (H + L) DyLight 800 (#SA5–10084, Thermo Fisher Scientific, RRID: AB_2556664). Membranes were imaged on a LI-COR Odyssey FC imager and analyzed with Image StudioTM software (LI-COR Biosciences). Coomassie staining of the gel was used to visualize total protein, which was used as a normalization control.

### 2.10 Statistics

All statistical analyses were performed using GraphPad Prism version 6.0 h software. When appropriate, a student's unpaired two-tailed *t*-test, a one-way ANOVA followed by a Tukey's multiple comparison test or a two-way ANOVA followed by a Sidak's multiple comparison test was used. Outliers were identified via the Grubbs' test and subsequently removed. For the Kaplan-Meier survival analysis, a log-rank test was used and survival curves were considered significantly different at *p* < 0.05 where *p < 0.05, **p < 0.01, ***p < 0.001 and ****p < 0.0001.

## 3 Results

### 3.1 Dysregulation of the GC-KLF15-BCAA Axis in Severe SMA Mice

**Fig. 1:**
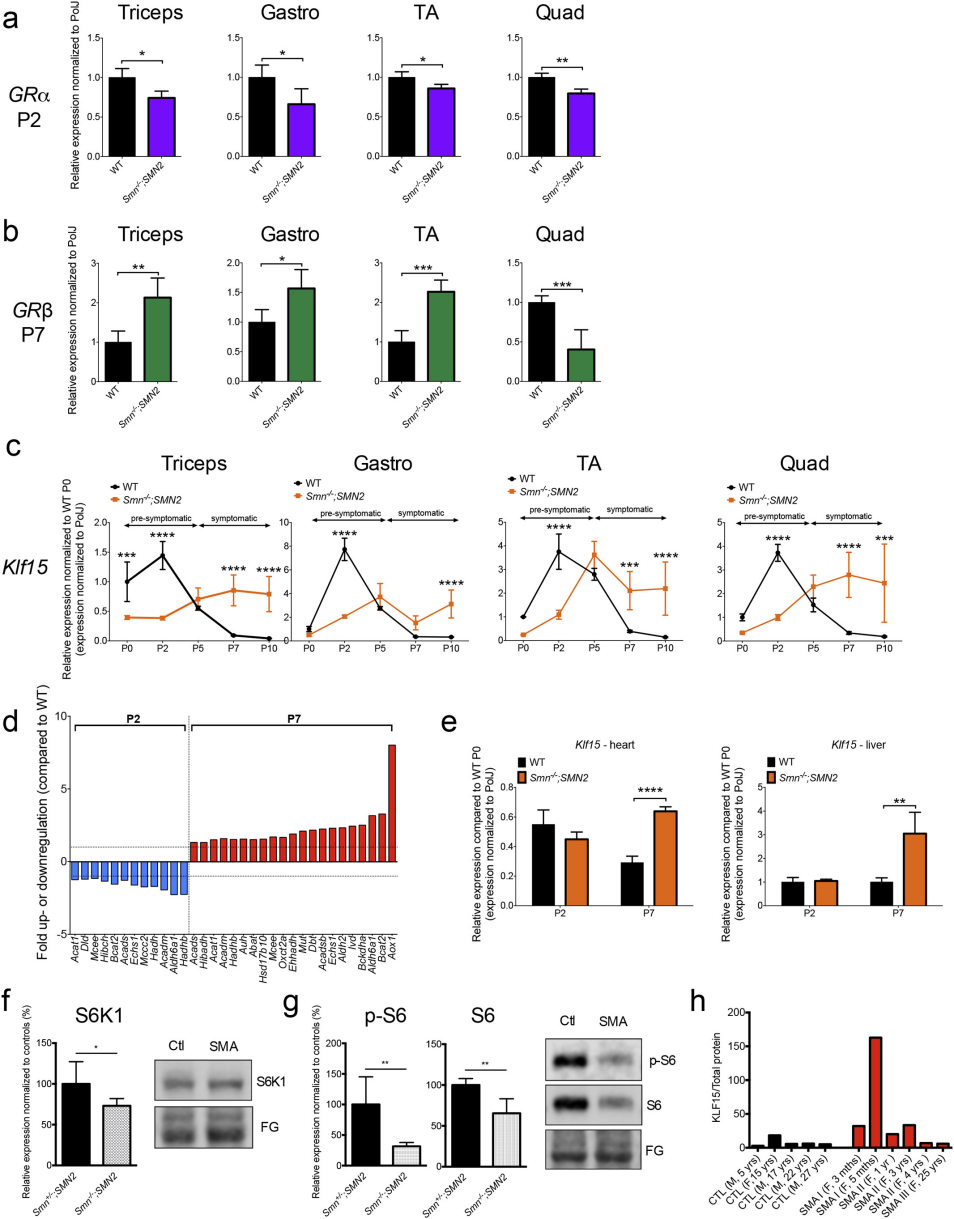
Dysregulation of the GC-KLF15-BCAA pathway in severe SMA mice and human SMA patients. a. qPCR analysis of *GRα* mRNA in four different skeletal muscles (*triceps brachii* (triceps), *gastrocnemius* (gastro), *tibialis anterior* (TA) and *quadriceps femoris* (quad)) of post-natal day (P) 2 *Smn*^*−/−*^*;SMN2* mice compared to WT animals. Data represent mean ± SD; *n* = 3–4 animals per group; two-tailed *t-test*; triceps: *p* = 0.0113; gastro: *p* = 0.0487; TA: *p* = 0.0176; quad: *p* = 0.0042. **b.** qPCR analysis of *GRβ* mRNA in four different skeletal muscles of P7 *Smn*^*−/−*^*;SMN2* mice compared to WT animals. Data represent mean ± SD; n = 3–4 animals per group; two-tailed *t-test*; triceps: *p* = 0.0075; gastro: *p* = 0.004; TA: *p* = 0.0352; quad: *p* = 0.0008. **c.** qPCR analysis of *Klf15* mRNA in four different skeletal muscles of *Smn*^*−/−*^*;SMN2* mice compared to WT animals at P0, P2, P5, P7 and P10. Data represent mean ± SD; n = 3–4 animals per group; two-way ANOVA; ***p < 0.001, ****p < 0.0001. **d.** BCAA metabolism effector genes (mRNA) dysregulated in triceps of P2 and P7 *Smn*^*−/−*^*;SMN2* animals compared to WT mice. Data represent fold up- or downregulation with *p* > 0.05. **e.** qPCR analysis of *Klf15* mRNA in heart and liver of P2 and P7 *Smn*^*−/−*^*;SMN2* mice compared to WT animals. Data represent mean ± SD, n = 3–4 animals per group, two-way ANOVA; **p < 0.01, ****p < 0.0001. **f.** Quantification of total S6 K1/total protein in triceps of P7 *Smn*^*−/−*^*;SMN2* mice compared to healthy littermates. Total protein was visualized with Fast Green (FG) stain. Images are representative immunoblots. Data represent mean ± SD, *n* = 5–7 animals per group, two-tailed *t-test*; *p* = 0.0325. **g.** Quantification of phosphorylated (p)-S6 and total S6/total protein in triceps of P7 *Smn*^*−/−*^*;SMN2* mice compared to healthy littermates. Total protein was visualized with Fast Green (FG) stain. Images are representative immunoblots. Data represent mean ± SD, n = 5–7 animals per group, two-tailed *t-test*; p-S6: *p* = 0.0024; total S6: *p* = 0.0024. **h.** Quantification of KLF15 protein/total protein in human gastrocnemius muscle samples from non-SMA control individuals and SMA Type I-III patients.

We first investigated the GC-KLF15-BCAA pathway in skeletal muscle from severe *Smn*^*−/−*^*;SMN2* SMA mice [44]. Muscles were selected based on their vulnerability to neuromuscular junction (NMJ) denervation (from most vulnerable to resistant: triceps > *gastrocnemius* (gastro) > *tibialis anterior* (TA) > *quadriceps femoris* (quad)) [45]. Muscles were harvested from *Smn*^*−/−*^*;SMN2* and wild type (WT) mice at several time-points during disease progression (post-natal day (P) 0: birth, P2: pre-symptomatic, P5: early symptomatic, P7: late symptomatic, P10: end stage). As GCs exert their influence on KLF15 via GR, we assessed expression of the two GR isoforms α and β [46] in muscle of P2 and P7 mice. GRα is thought to be a key mediator of GC-dependent target gene transactivation, while GRβ inhibits GRα and induces GC resistance [47]. Interestingly, we observed a significant downregulation of *GRα* mRNA in P2 and upregulation of *GRβ* mRNA in P7 *Smn*^*−/−*^*;SMN2* mice compared to WT (Fig. 1a, b), with the exception of P7 quad where *GRβ* is significantly decreased in SMA animals. Both, *GRβ and GRα* mRNA levels are not significantly different between *Smn*^*−/−*^*;SMN2* and WT mice at P2 and P7, respectively (Supplementary Fig. 1a, b), with the exception of P7 gastro where *GRα* is significantly decreased in SMA animals.

We next examined the expression profile of *Klf15* mRNA during disease progression and found the same pattern in all four muscles: decreased levels in P2 and increased levels in P7 *Smn*^*−/−*^*;SMN2* mice compared to WT animals (Fig. 1c).

Interestingly, the peak expression of *Klf15* mRNA in WT muscles occurs at P2 while in SMA muscles, it is observed at P5 or later, reflecting a potential developmental delay, which has previously been reported for myogenic regulatory factors (MRFs) [27,48]. To evaluate if similar developmental delays also occurred for *GRa* and *GRb* mRNA expression, we further determined their expression profiles at all time-points during disease progression. Interestingly, we observed peak levels at P2 for both *GRa* and *GRb* mRNAs for most WT muscles (Supplementary Fig. 2). However, in SMA muscles, *GRa* and *GRb* mRNA levels either remained similar throughout or displayed a slight downregulation/upregulation in symptomatic stages (Supplementary Fig. 2). Thus, the expression profiles of *GRα* and *GRβ* mRNAs are distinct from that of *Klf15* mRNA.

We next wanted to determine if the increased *Klf15* mRNA expression corresponded to previously reported differential Smn expression during neonatal muscle development in a different severe SMA mouse model [49]. We find that *Smn* mRNA levels do not significantly change in WT muscles from P0 to P10, with the exception of a significant increase in P2 quad (Supplementary Fig. 3). Our findings are consistent with another study demonstrating that Smn protein levels in hindlimb muscles from WT animals are relatively high and similar from P0 to P10, followed by a dramatic decrease from P10 onwards [27].

Seeing as aberrant *Klf15* expression may alter BCAA metabolism, we performed commercially available Amino Acid Metabolism PCR arrays on P2 and P7 triceps (Supplementary Table 1). We observed that the expression of a number of effectors of BCAA metabolism were significantly downregulated in P2 and upregulated in P7 *Smn*^*−/−*^*;SMN2* mice compared to WT animals (Fig. 1d). These included *Bcat2 mRNA,* the major catabolic enzyme of BCAAs [6], previously shown to be regulated by KLF15 activity [5]. We next evaluated *Klf15* mRNA expression in heart and liver of P2 and P7 animals, two metabolic tissues highly influenced by KLF15 activity [4,50]. *Klf15* mRNA levels were unchanged in tissues from P2 mice but were significantly increased in heart and liver from P7 *Smn*^*−/−*^*;SMN2* mice compared to WT animals (Fig. 1e). Therefore, our results suggest that the decreased activity of the GC-KLF15-BCAA pathway in pre-symptomatic SMA mice is limited to skeletal muscle, whereas the increased activity in symptomatic SMA animals may be a more widespread phenomenon.

Maintenance of muscle function and mass throughout life depends on the balance between protein synthesis and degradation regulated by mammalian target of rapamycin (mTOR) [51]. Amino acid availability, particularly the BCAA leucine, stimulates mTOR complex 1 (mTORC1) protein synthesis in muscle [51]. KLF15 thus interferes with mTOR protein synthesis by promoting BCAT2 activity and subsequent degradation of leucine [12]. We therefore investigated mTOR activity in skeletal muscle (triceps) of P7 *Smn*^*−/−*^*;SMN2* mice and control littermates, which, to the best of our knowledge, has not yet been performed in the Taiwanese SMA mouse model. Ribosomal protein S6 kinase beta-1 (S6 K1) is phosphorylated (p) following mTOR activation and subsequently directly phosphorylates the S6 ribosomal protein (S6) [51]. As direct downstream effectors, both S6 K1 and S6 were therefore used to assess mTOR activity. Immunoblot analysis revealed that protein levels of S6 K1, p-S6 and S6 are significantly downregulated in triceps of *Smn*^*−/−*^*;SMN2* mice compared to healthy littermates (Fig. 1f, g). Thus, the mTOR-dependent protein synthesis is significantly downregulated in SMA muscle, which could be directly linked to the upregulated *Klf15* mRNA levels.

We also obtained human muscle biopsies (gastrocnemius) from control non-SMA individuals and SMA Type I-III patients (most severe to less severe: Type I > Type II > Type III) of varying ages (3 mths-27 yrs). Western blot analysis of KLF15 protein revealed a trend for increased KLF15 levels in SMA muscle samples (Fig. 1h, Supplementary Fig. 4). These protein expression patterns therefore prompt further detailed studies of GC-KLF15-BCAA signaling in SMA patients.

Finally, we wanted to assess if dysregulation of *Klf15*, *GRα* and *GRβ* mRNAs was linked to SMN levels. To do so, we used muscle from *Smn*^*−/−*^*;SMN2* mice that received facial vein injections at P0 and P2 of our previously published Pip6a-phosphorodiamidate oligomer (PMO) compound that promotes full length SMN production from the human *SMN2* gene [39]. P7 TAs from Pip6a-PMO-treated *Smn*^*−/−*^*;SMN2* mice were compared to age-matched tissues from WT animals as well as untreated and Pip6a-scrambled-treated *Smn*^*−/−*^*;SMN2* mice. Interestingly, we find that *Klf15* mRNA is significantly reduced in in both Pip6a-PMO and Pip6a-scrambled-treated muscles (Supplementary Fig. 5a), suggesting an SMN-independent normalization of *Klf15* mRNA levels. Indeed, our combined qPCR, transcriptomics and proteomics analysis of these tissues shows that while the Pip6a-scrambled compound does not increase *FL SMN2* mRNA expression, numerous transcripts and proteins are significantly differentially regulated compared to muscle from untreated SMA mice (data not shown). The aberrant expression of *Klf15* mRNA and its restoration in Pip6a-treated muscle may therefore be more related to overall muscle and whole-body metabolic state and activity [52,53]. Both Pip6a-PMO and Pip6a-scrambled had no normalization effects on *GRα* mRNA levels (Supplementary Fig. 5b) while similar SMN-independent effects were observed for *GRβ* mRNA levels (Supplementary Fig. 5c).

### 3.2 Altered Diurnal Expression of the GC-KLF15-BCAA Pathway in Severe SMA Mice

**Fig. 2:**
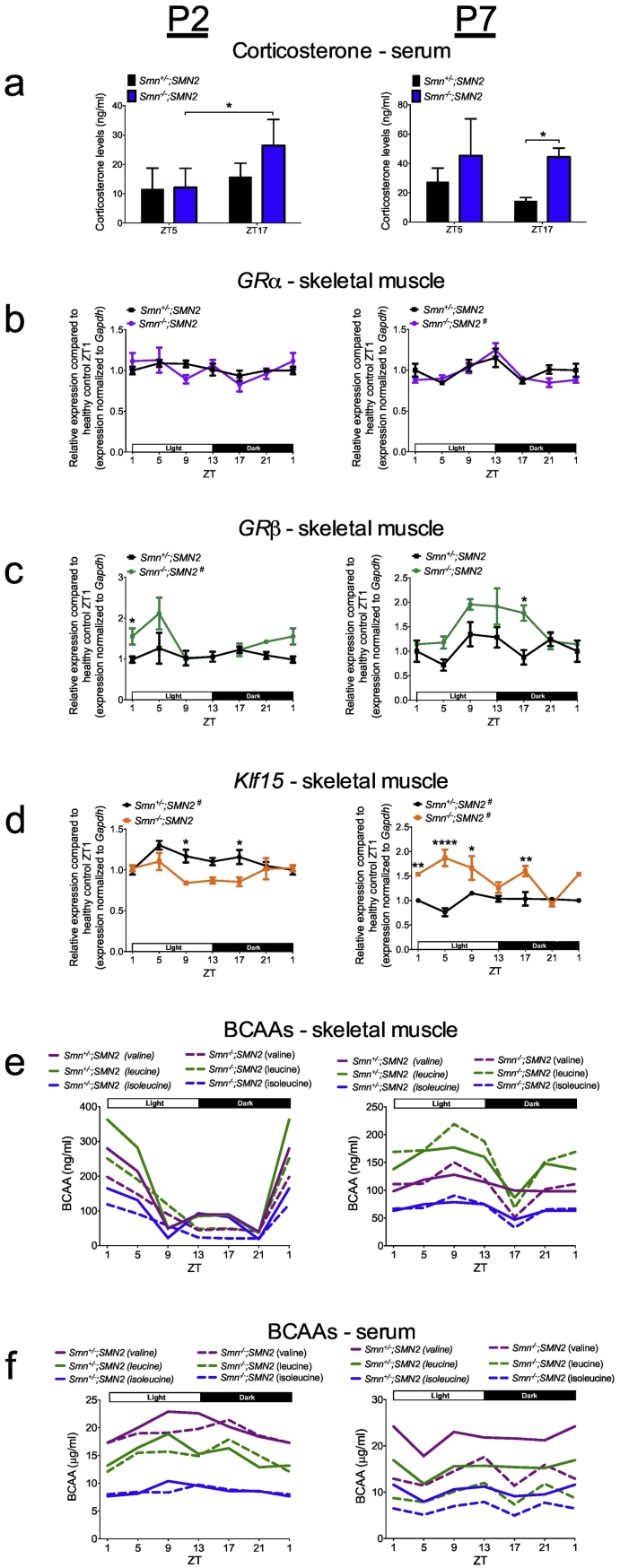
Circadian rhythmicity of the GC-KLF15-BCAA axis is dysregulated in severe SMA mice. a. Corticosterone levels in serum of post-natal day (P) 2 and P7 *Smn*^*−/−*^*;SMN2* mice compared to healthy control littermates at the Zeitgeber time (ZT) 5 and ZT17. Data represent mean ± SD; n = 3–4 animals per group, two-way ANOVA; *p < 0.05 **b.** qPCR analysis of diurnal expression of *GRα* mRNA in the *tibialis anterior* (TA) of P2 and P7 *Smn*^*−/−*^*;SMN2* mice compared to healthy controls. **c.** qPCR analysis of diurnal expression of *GRβ* mRNA in the TA of P2 and P7 *Smn*^*−/−*^*;SMN2* mice compared to healthy controls. **d.** qPCR analysis of diurnal expression of *Klf15* mRNA in the TA of P2 and P7 *Smn*^*−/−*^*;SMN2* mice compared to healthy controls. **b-d:** Data represent mean ± SD; n = 3–5 animals per group, two-way ANOVA; *p < 0.05, **p < 0.01, ****p < 0.0001; # indicates cycling ZT1 data is duplicated. **e.** Levels of the BCAAs valine, leucine and isoleucine in triceps of P2 and P7 *Smn*^*−/−*^*;SMN2* and healthy controls. **f.** Levels of the BCAAs in serum of P2 and P7 *Smn*^*−/−*^*;SMN2* and healthy control animals. **e–f:** each data point represents the pooling of 5–15 animals**.**

All components of the GC-KLF15-BCAA pathway display functional and regulatory circadian expression patterns [5,54]. As our analysis so far corresponds to a single time-point during a 24 h period, we next assessed the circadian rhythmicity of the GC-KLF15-BCAA axis in SMA mice. Upon pairing, breeding pairs were entrained to a 12 h light:12 h dark cycle (LD12:12) and ensuing litters maintained in that environment. Serum, TA and triceps were harvested from P2 and P7 *Smn*^*−/−*^*;SMN2* and *Smn*^*+/−*^*;SMN2* control littermates every 4 h (Zeitgeber time, ZT) over a 24 h period (ZT0 = 8 am, ZT1 = 9 am, ZT5 = 1 pm, ZT9 = 5 pm, ZT13 = 9 pm, ZT17 = 1 am, ZT21 = 5 am). Corticosterone (major mouse GC) levels in serum were measured by ELISA at ZT5 (day) and ZT17 (night) and show significantly dysregulated levels in *Smn*^*−/−*^*;SMN2* mice defined by elevated release in the dark phase compared to control littermates (Fig. 2a). Assessment of *GR* gene expression in TA shows that the diurnal pattern of *GRα* mRNA is relatively similar between *Smn*^*−/−*^*;SMN2* mice and control littermates (Fig. 2b). However, *GRβ* mRNA displays significant changes in amplitude, whereby we observe similar oscillation patterns but with differential expression at specific ZTs in both P2 and P7 *Smn*^*−/−*^*;SMN2* mice compared to control littermates (Fig. 2c). The overall upregulation of *GRβ* known to mediate metabolic GC resistance [46,47] may be a compensatory mechanism to counteract the aberrant GC regulation identified in Fig. 2a. Analysis of diurnal expression of *Klf15* mRNA also shows changes in amplitude with a general downregulation in P2 and upregulation in P7 *Smn*^*−/−*^*;SMN2* mice compared to control littermates (Fig. 2d). The fact that *GRα/β* and *Klf15* levels are similar between groups at certain ZTs could suggest that the defect lies in circadian regulation and not overall expression as well as highlights discrepancies in data interpretation that can arise when circadian effectors are analyzed at one single time-point.

To determine if circadian BCAA metabolism was impacted, valine, leucine and isoleucine levels were measured by high-performance liquid chromatography (HPLC) in serum and triceps of P2 and P7 *Smn*^*−/−*^*;SMN2* mice and control littermates over a 24 h period. In muscle, we report diurnal cycling of BCAAs in P2 and P7 mice with changes in phase (distinct oscillation patterns) and amplitude at both time-points between *Smn*^*−/−*^*;SMN2* mice and control littermates (Fig. 2e), with the exception of isoleucine at P7. Similar observations were made when looking at serum BCAA levels, where the 24 h cycling behaviour in P2 and P7 *Smn*^*−/−*^*;SMN2* mice demonstrates differences in phase and amplitude compared to control littermates (Fig. 2f), with the exception of isoleucine at P2. Of particular interest is the generalized depletion of all BCAAs in the serum of P7 *Smn*^*−/−*^*;SMN2* animals (Fig. 2f), which may reflect the high use in skeletal muscle due to increased *Klf15* expression at the same time-point (Fig. 2d).

Finally, to assess if aberrant circadian expression of *Klf15* mRNA was specific to skeletal muscle, we evaluated its rhythmicity in various metabolic tissues (white adipose tissue (WAT), brown adipose tissue (BAT), liver and heart) and spinal cord (SC) from P2 and P7 *Smn*^*−/−*^*;SMN2* mice and control littermates (Supplementary Fig. 6). We find changes in phase and amplitude in all P2 tissues except for SC while at P7, all tissues display significant phase and amplitude alterations highlighted by an overall significant increase in *Klf15* mRNA expression. These systemic alterations in *Klf15* expression could further influence the serum depletion of BCAAs observed in P7 *Smn*^*−/−*^*;SMN2* mice (Fig. 2f). Combined, our results demonstrate a dysregulated circadian regulation of the GC-KLF15-BCAA pathway in *Smn*^*−/−*^*;SMN2* mice, which is specific to skeletal muscle in the pre-symptomatic stage and evolves to a whole-body phenomenon as disease progresses.

**Fig. 3:**
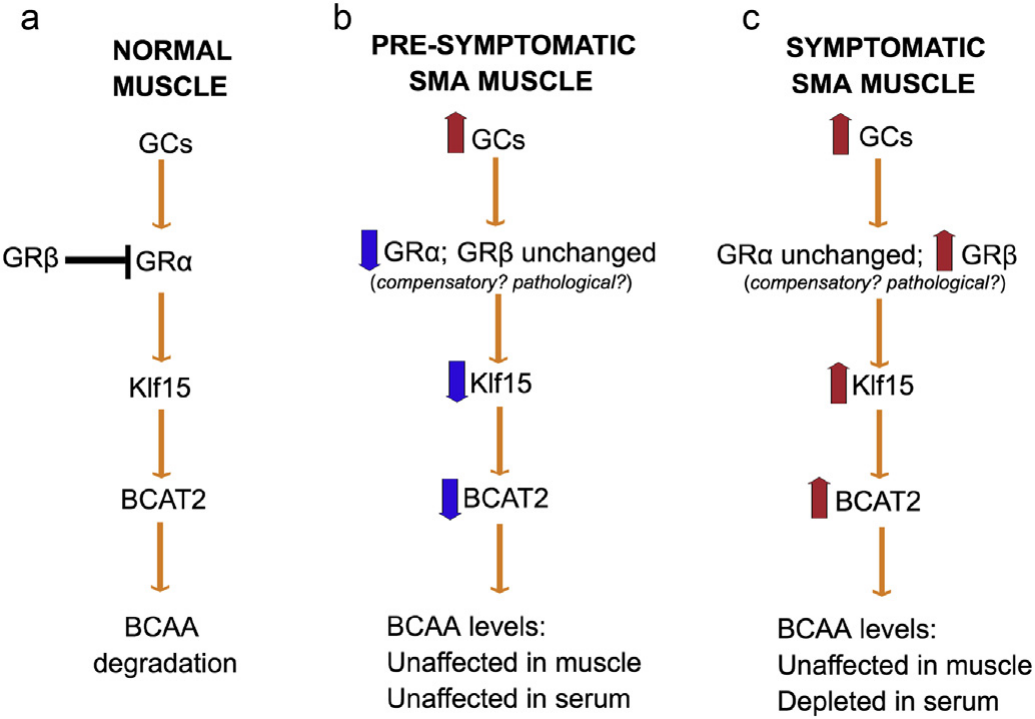
Schematic summarizing the activity of the glucocorticoid (GC)- glucocorticoid receptor (GR, α and β)-Klf15-BCAT2-branched-chain amino acid (BCAA) signaling cascade in normal muscle (a), pre-symptomatic muscle from *Smn*^*−/−*^*;SMN2* SMA mice (b) and symptomatic muscle from *Smn*^*−/−*^*;SMN2* SMA mice (c).

Altogether, our analysis of GC-KLF15-BCAA signaling during disease progression (Fig. 1) and over a 24 h period (Fig. 2), demonstrate an overall downregulated activity in pre-symptomatic muscle and upregulated activity in symptomatic muscle (Fig. 3), which could potentially have distinct effects on the development and/or maintenance of muscle and metabolic pathologies in SMA.

### 3.3 Modulating Upstream GC-KLF15-BCAA Signaling With Prednisolone Improves Phenotype of Severe SMA Mice

**Fig. 4:**
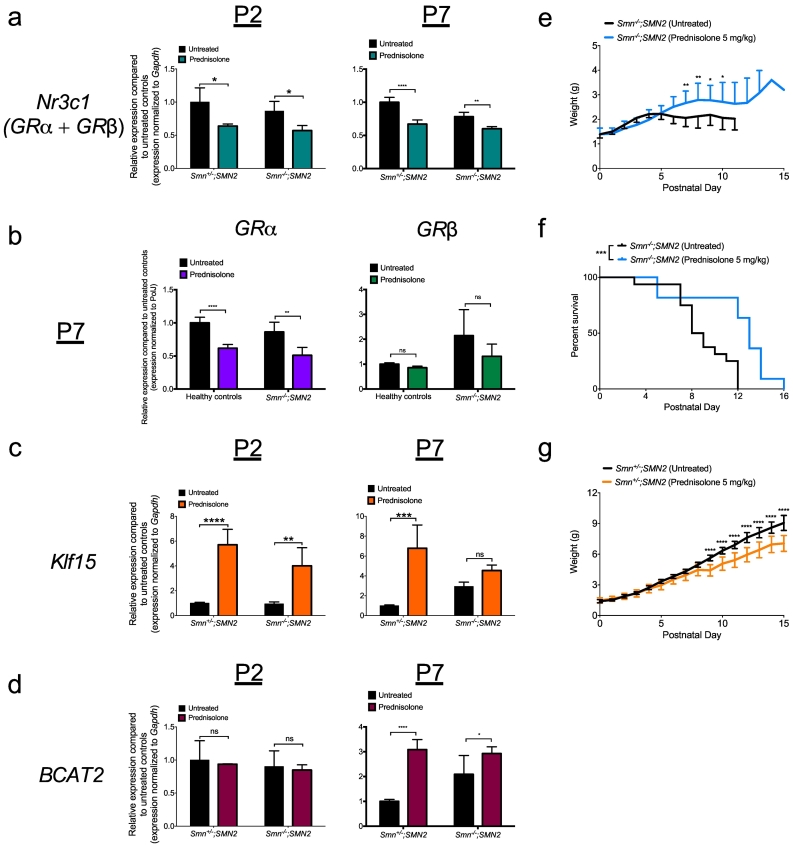
Prednisolone treatment improves disease phenotypes in severe SMA mice. qPCR analysis of **(a)***Nr3c1***,** (**b**) *GRα* and *GRβ* (**c**) *Klf15* and (**d**) *Bcat2* mRNAs in *tibialis anterior* (TA) muscle of post-natal day (P) 2 and P7 untreated and prednisolone-treated *Smn*^*−/−*^*;SMN2* mice and control littermates. **a-d:** Data represent mean ± SD; n = 3–4 animals per group; two-way ANOVA; *p < 0.05, **p < 0.01, ****p < 0.0001; ns = not significant. **e.** Weight curves of prednisolone-treated *Smn*^*−/−*^*;SMN2* mice vs. untreated animals. Data represent mean ± SD; *n* = 10–16 animals per group; *p < 0.05, **p < 0.01. **f.** Lifespan of prednisolone-treated *Smn*^*−/−*^*;SMN2* mice vs. untreated animals. Data represent Kaplan-Meier curves; n = 10–16 animals per group; Log-rank (Mantel-Cox) test; *p* = 0.0009. **g.** Weight curves of prednisolone-treated healthy controls vs. untreated animals. Data represent mean ± SD; *n* = 9–18 animals per group; ****p < 0.0001.

We next set out to determine if aberrant regulation of *Klf15* mRNA in *Smn*^*−/−*^*;SMN2* mice has a physiological impact on major disease phenotypes. Firstly, to counteract the muscle-specific downregulation of *Klf15* mRNA in pre-symptomatic *Smn*^*−/−*^*;SMN2* mice (Fig. 1c, 2d), we used the pharmacological compound prednisolone, a synthetic GC previously demonstrated to specifically induce *Klf15* mRNA expression [55,56]. We administered prednisolone by gavage to *Smn*^*−/−*^*;SMN2* mice and control littermates every 2 days starting at P0. A dose-response assessment of prednisolone (2.5, 5 and 10 mg/kg) determined the optimal dose of 5 mg/kg (Supplementary Fig. 7). We firstly validated the direct action of prednisolone on the GC-KLF15-BCAA pathway in muscle of P2 and P7 *Smn*^*−/−*^*;SMN2* mice and control littermates. We found that the expression of total *GR* mRNA (*Nr3c1, GRα + GRβ*) is significantly reduced in P2 and P7 prednisolone-treated mice compared to untreated animals (Fig. 4a), most likely attributed to a GC-mediated downregulation of *GR* gene transcription [57]. Further analysis revealed that this downregulation was specifically attributed to a decreased expression of *GRα* mRNA as *GRβ* mRNA levels were unchanged (Fig. 4b). As total *GRβ* mRNA levels are consistently significantly less abundant than *GRα* mRNA levels, the latter will therefore have greater impact on total *GR* (*Nr3c1*) mRNA levels. As expected, *Klf15* mRNA levels are significantly enhanced in P2 *Smn*^*−/−*^*;SMN2* mice and control littermates treated with prednisolone compared to untreated animals (Fig. 4c). At P7 however, *Klf15* mRNA is significantly upregulated in prednisolone-treated control littermates compared to untreated mice while no changes are seen in prednisolone-treated *Smn*^*−/−*^*;SMN2* mice (Fig. 4c), suggesting that Klf15 signaling in SMA mice becomes less responsive to GCs as disease progresses, potentially as a result of the increased expression of *GRβ* (Fig. 1b, 2c). Finally, mRNA levels of the direct transcriptional target of KLF15, *Bcat2* [4] were unchanged in P2 *Smn*^*−/−*^*;SMN2* mice and control littermates (Fig. 4d), most likely reflecting the delayed increase of *Bcat2* expression following *Klf15* induction [12]. Indeed, we detected a significant upregulation of *Bcat2* mRNA in muscles from P7 prednisolone-treated *Smn*^*−/−*^*;SMN2* mice and control littermates compared to untreated animals (Fig. 4d).

Importantly, we demonstrate that *Smn*^*−/−*^*;SMN2* animals display a significant increased weight gain (Fig. 4e) and an enhanced lifespan after prednisolone administration (Fig. 4f). In contrast, control littermates mice show a significant weight reduction when treated with prednisolone (Fig. 4g), which could be attributed to the typical muscle wasting effect of prolonged exposure to GCs [58]. Thus, our results suggest that modulating GC-KLF15-BCAA signaling with a synthetic GC is a valid therapeutic strategy for SMA.

### 3.4 Modulating GC-KLF15-BCAA Signaling With Prednisolone Improves Phenotype of Intermediate SMA Mice

**Fig. 5:**
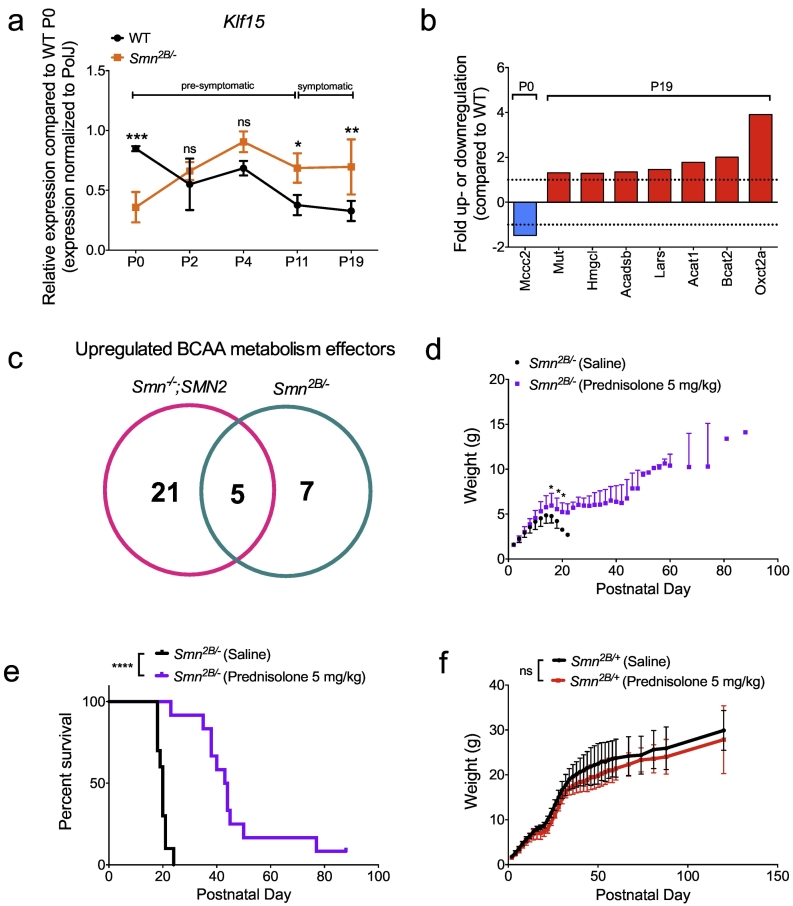
Dysregulation of the GC-KLF15-BCAA pathway in intermediate SMA mice and prednisolone-induced phenotypic improvements. a. qPCR analysis of *Klf15* mRNA in *tibialis anterior* (TA) of *Smn*^*2B/−*^ mice compared to WT animals at different ages (post-natal day (P) 0, P2, P4, P11 and P19). Data represent mean ± SD; *n* = 4 animals per group; two-way ANOVA; *p < 0.05, **p < 0.01, ***p < 0.001; ns = not significant. **b.** BCAA metabolism effector genes (mRNA) dysregulated in TAs of pre- and symptomatic *Smn*^*2B/−*^ mice compared to WT animals. Data represent fold up- or downregulation with *p* > 0.05. **c.** Venn diagram demonstrating the number of upregulated BCAA metabolism effectors in TAs of symptomatic *Smn*^*−/−*^*;SMN2* and *Smn*^*2B/−*^ mice. **d.** Weight curves of prednisolone-treated *Smn*^*2B/−*^ mice vs. saline-treated animals. Data represent mean ± SD; n = 10–12 animals per group; two-way ANOVA; *p < 0.05; ns = not significant. **e.** Lifespan of prednisolone-treated *Smn*^*2B/−*^ mice vs. saline-treated animals. Data represent Kaplan-Meier curves; n = 10–12 animals per group; Log-rank (Mantel-Cox) test; *p* < 0.0001. **f.** Weight curves of *Smn*^*2B/+*^ mice treated with prednisolone or saline. Data represent mean ± SD; *n* = 7–10 animals per group; two-way ANOVA; ns = not significant.

To investigate whether the observed dysregulation in the GC-KLF15-BCAA axis is present in other SMA mouse models, we repeated key experiments in the intermediate *Smn*^*2B/−*^ mice [59,60]. Similar to the severe SMA mouse model, *Smn*^*2B/−*^ mice display a significant reduction in *Klf15* mRNA expression in pre-symptomatic muscle (TA) followed by a significant upregulation during disease progression compared to age-matched WT animals (Fig. 5a). Using commercially available Amino Acid Metabolism PCR arrays (Supplementary Table 1), we also demonstrate perturbed expression of BCAA metabolism effectors, particularly in symptomatic *Smn*^*2B/−*^ mice, where several genes are significantly increased compared to WT animals (Fig. 5b). Interestingly, we noted a comparable upregulation of *Bcat2, Oxct2a, Acat1, Acadsb* and *Mut* mRNAs in symptomatic severe *Smn*^*−/−*^*;SMN2* and intermediate *Smn*^*2B/−*^ SMA mice (Fig. 5c). Importantly, we also administered prednisolone (5 mg/kg) by gavage to *Smn*^*2B/−*^ mice and *Smn*^*2B/+*^ control littermates every 2 days from P0 to P20. This dosing regimen had a significant beneficial effect on weight gain (Fig. 5d) and led to an enhanced lifespan (Fig. 5e) of treated *Smn*^*2B/−*^ mice compared to saline-treated animals. Prednisolone had no significant impact on weight of *Smn*^*2B/+*^ control littermates (Fig. 5f). We thus show a dysregulated *Klf15* pathway in muscle of two distinct SMA mouse models, and importantly demonstrate that modulating the GC-KLF15 signaling cascades via administration of prednisolone improves weight and survival in both *Smn*^*−/−*^*;SMN2* and *Smn*^*2B/−*^ mice.

### 3.5 Prednisolone Improves Neuromuscular Phenotype of Severe SMA Mice

**Fig. 6:**
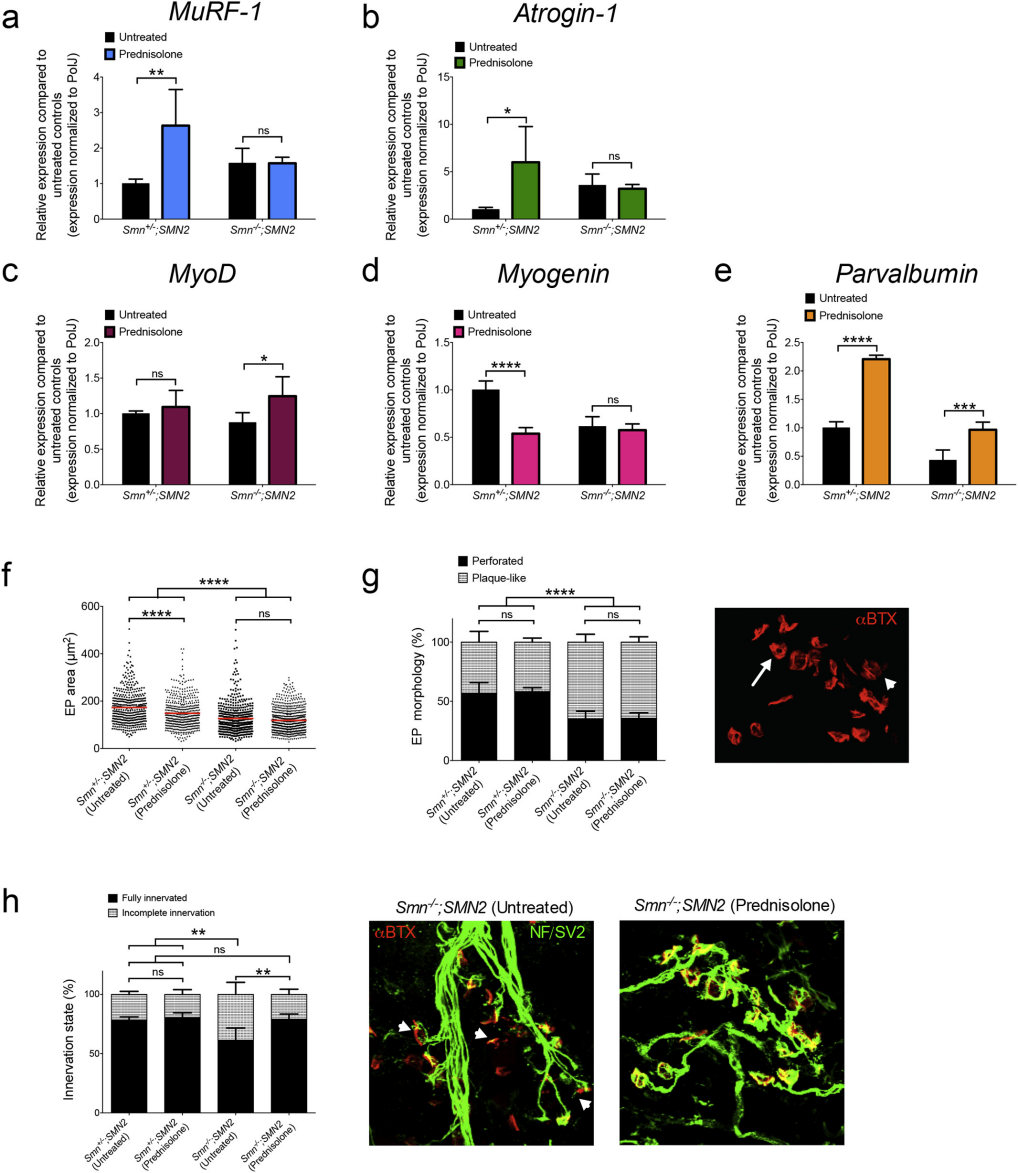
Prednisolone treatment improves neuromuscular phenotypes in severe SMA mice. *Smn*^*−/−*^*;SMN2* mice and healthy littermates were treated with 5 mg/kg prednisolone every second day beginning from P0. qPCR analysis of (**a**) *MuRF-1*, (**b**) *atrogin1*, (**c**) *MyoD*, (**d**) *myogenin* and (**e**) *parvalbumin* mRNAs in triceps of P7 *Smn*^*−/−*^*;SMN2* mice and healthy littermates treated with prednisolone compared to untreated animals. **a-e:** Data represent mean ± SD; n = 3–4 animals per group; two-way ANOVA; *p < 0.05, **p < 0.01, ***p < 0.001, ****p < .0001; ns = not significant. **f.** Motor endplate area in TAs of untreated and prednisolone-treated P7 *Smn*^*−/−*^*;SMN2* mice and healthy littermates. Data represent scatter plot ± SD; *n* = 424–711 endplates from 4 animals per group; one-way ANOVA; ****p < 0.0001; ns = not significant. **g.** Quantitative analysis of motor endplate morphology (plaque-like or perforated) in TAs of untreated and prednisolone treated P7 *Smn*^*−/−*^*;SMN2* mice and healthy littermates. Representative image of endplates where arrow indicates perforated and arrowhead indicates plaque-like. **h.** Quantitative analysis of the innervation status of motor endplates in TAs of untreated and prednisolone-treated P7 *Smn*^*−/−*^*;SMN2* mice and healthy control littermates. Representative image of NMJs from untreated and prednisolone-treated *Smn*^*−/−*^*;SMN2* mice where arrowhead indicates incomplete innervation. **g-h.** Data represent mean ± SD; n = 4 animals per group; two-way ANOVA; **p < 0,01; ns = not significant.

Having shown that GC treatment ameliorates SMA disease progression in intermediate and severe SMA mouse models, we further analyzed the effects of prednisolone on neuromuscular pathology in P7 *Smn*^*−/−*^*;SMN2* mice and control littermates. We first assessed the effect of prednisolone on the expression of *MuRF-1* and *atrogin-1* mRNAs, ubiquitin ligases involved in muscle atrophy [61] and typically induced by chronic administration of GCs [62]. Expression of mRNA of both atrogenes is significantly increased in treated control littermates compared to untreated control animals while no differences are found between prednisolone-treated and untreated *Smn*^*−/−*^*;SMN2* mice (Fig. 6a, b). The GC induction of *MuRF-1* and *atrogin-1* mRNAs in healthy animals only may explain the reduced weights specifically observed in prednisolone-treated control littermates (Fig. 4g).

We next investigated the impact of prednisolone on expression of *MyoD*, *myogenin* and *parvalbumin* mRNAs, determinants of muscle health previously involved in SMA muscle pathology [27,43,48]. MyoD and myogenin are myogenic regulatory factors (MRFs) that modulate commitment to muscle lineage and muscle-specific gene expression [63]. Parvalbumin is a marker for neuromuscular perturbations as its expression is decreased in denervated muscles [64] and in symptomatic muscle of SMA patients and *Smn*^*−/−*^*;SMN2* mice [43]. We found that *MyoD* mRNA expression is significantly enhanced in muscle of prednisolone-treated *Smn*^*−/−*^*;SMN2* mice compared to untreated animals while GCs did not impact *MyoD* mRNA levels in control littermates (Fig. 6c). In contrast, prednisolone caused a significant reduced expression of *myogenin* mRNA in control littermates while no significant changes in expression were observed between treated and untreated *Smn*^*−/−*^*;SMN2* mice (Fig. 6d). The downregulation of *myogenin* mRNA in control littermates treated with prednisolone may reflect the increased atrophy signaling [65] (Fig. 6a, b) and weight loss (Fig. 4g) specifically observed in this experimental cohort. Interestingly, comparison of *parvalbumin* mRNA expression in *Smn*^*−/−*^*;SMN2* mice and control littermates shows that prednisolone significantly increases *parvalbumin* mRNA levels in both groups (Fig. 6e), which in SMA mice has previously been associated with improved muscle health [43].

We then wanted to determine if molecular changes generated by prednisolone administration would ameliorate the denervation pathology and developmental defects at the NMJ [66]. Assessment of NMJs in TAs of P7 animals showed a significant reduction in motor endplate area of untreated *Smn*^*−/−*^*;SMN2* mice compared to untreated control littermates, which remained unchanged in prednisolone-treated *Smn*^*−/−*^*;SMN2* animals (Fig. 6f). Interestingly, treated control animals display a significantly smaller endplate area compared to untreated control littermates (Fig. 6f), again depicting the adverse impact of prednisolone on muscle of control animals. Next, we examined endplate morphology by distinguishing between plaque-like and perforated endplates, whereby in the maturation process, their shape changes from plaque-like at P0, to perforated at P5, and finally to pretzel-like at P10 [67]. We find that both treated and untreated *Smn*^*−/−*^*;SMN2* mice display significantly more immature appearing endplates compared to treated and untreated control littermates (Fig. 6g). Finally, we quantified the innervation status of endplates and demonstrate that prednisolone significantly increases the number of fully innervated NMJs in *Smn*^*−/−*^*;SMN2* mice compared to untreated animals (Fig. 6h). Interestingly, prednisolone has previously been demonstrated to play a beneficial role in the pre-synaptic compartment of the NMJ, by preventing a drug-induced neuromuscular blockade [68]. The improved NMJ innervation may also be associated with the increased *parvalbumin* expression observed in muscle of prednisolone-treated *Smn*^*−/−*^*;SMN2* mice (Fig. 6e), known to reflect the denervation status of muscle [64]. Taken together, our molecular and histopathological analyses reveal a differential response to prednisolone between *Smn*^*−/−*^*;SMN2* mice and control littermates. Muscles from control animals undergo a GC-induced atrophy, while this pathway is not activated in SMA mice. Rather, *Smn*^*−/−*^*;SMN2* muscles show a myogenic response and a restoration of fully innervated endplates, which potentially explain the ameliorated phenotype of prednisolone-treated *Smn*^*−/−*^*;SMN2* mice.

### 3.6 Synergistic Effect of Prednisolone and *Klf15* Overexpression on Muscle Pathology of SMA Mice

**Fig. 7:**
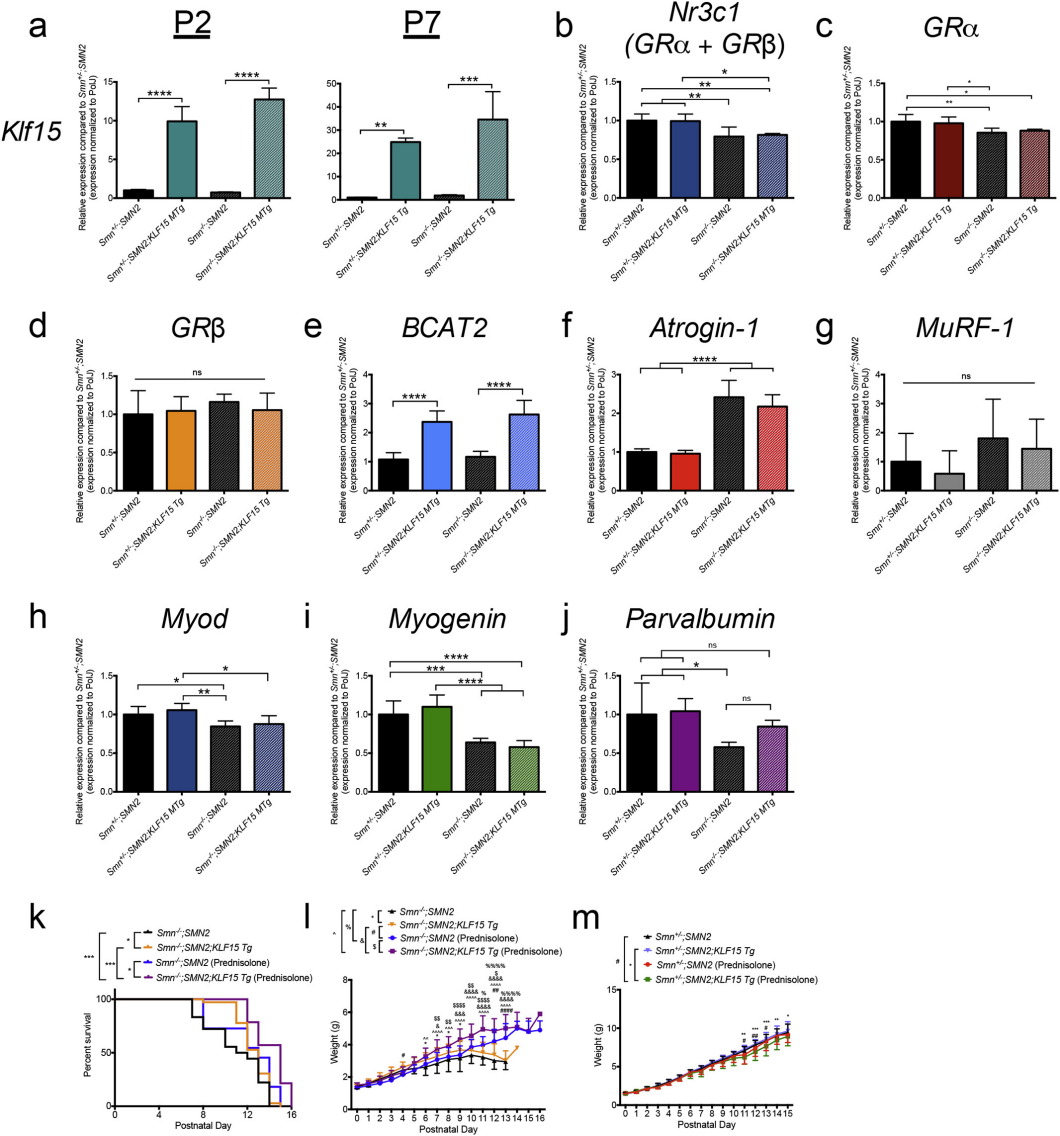
Synergistic effects of *Klf15* overexpression and prednisolone on disease phenotypes of severe SMA mice. a. qPCR analysis of *Klf15* mRNA in skeletal muscle (quadriceps) from post-natal day (P) 2 and 7 *Smn*^*−/−*^*;SMN2*, *Smn*^*+/−*^*;SMN2*, *Smn*^*−/−*^*;SMN2;KLF15 MTg* and *Smn*^*+/−*^*;SMN2;KLF15* MTg mice. Data represent mean ± SD; n = 3–8 animals per group; two-way ANOVA; **p < 0.01, ***p < 0.001, ****p < 0.0001. qPCR analysis of (**b**) *Nr3c1*, (**c**) *GRα*, (**d**) *GRβ*, (**e**) *Bcat2*, (**f**) *atrogin-1***,** (**g**) *MuRF-1*, (**h**) *Myod*, (**i**) *myogenin* and (**j**) *parvalbumin* mRNAs in quadriceps of P7 *Smn*^*−/−*^*;SMN2*, *Smn*^*+/−*^*;SMN2*, *Smn*^*−/−*^*;SMN2;KLF15 MTg* and *Smn*^*+/−*^*;SMN2;KLF15* MTg mice. **b-j**: Data represent mean ± SD; *n* = 6–8 animals per group; one-way ANOVA; *p < 0.05, **p < 0.01, ***p < 0.001, ****p < 0.0001, ns = not significant. **k.** Lifespan of untreated and prednisolone-treated *Smn*^*−/−*^*;SMN2* and *Smn*^*−/−*^*;SMN2;KLF15 MTg* mice. Data represent Kaplan-Meier curves; *n* = 11–36 animals per group; Log-rank test; *p < 0.05, ***p < 0.001. **l.** Weight curves of untreated and prednisolone-treated *Smn*^*−/−*^*;SMN2* and *Smn*^*−/−*^*;SMN2;KLF15 MTg* mice. Data represent mean ± SD; n = 7–10 animals per group; two-way ANOVA; *p < 0.05, **p < 0.01, ***p < 0.001, ****p < 0.0001. **m.** Weight curves of untreated and prednisolone-treated *Smn*^*+/−*^*;SMN2* and *Smn*^*+/−*^*;SMN2;KLF15 MTg* mice. Data represent mean ± SD; n = 7–10 animals per group; two-way ANOVA; *p < 0.05, **p < 0.01, ***p < 0.001, ****p < 0.0001.

To better evaluate the impact of prednisolone-dependent *Klf15* induction in SMA animals, we generated transgenic *Smn*^*−/−*^*;SMN2* mice that overexpress *Klf15* specifically in skeletal muscle by crossing the SMA line with the previously described *KLF15 MTg* mice [55]. The ensuing F1 litters generated *Smn*^*−/−*^*;SMN2*, *Smn*^*+/−*^*;SMN2*, *Smn*^*−/−*^*;SMN2;KLF15 MTg* and *Smn*^*+/−*^*;SMN2;KLF15* MTg mice that were on a mixed background of C57BL/6 (*KLF15 MTg* line) and FVB/N (*Smn*^*−/−*^*;SMN2* line). We first assessed the activity of the skeletal muscle specific enhancer/promoter (muscle creatine kinase (MCK)) driving *Klf15* expression in P2 and P7 tissues. We found a significant increased expression of *Klf15* mRNA in the quadriceps muscle of both P2 and P7 *Smn*^*−/−*^*;SMN2;KLF15 MTg* and *Smn*^*+/−*^*;SMN2;KLF15* MTg mice (Fig. 7a). To determine how this upregulation of *Klf15* affected GC-KLF15-BCAA signaling, we analyzed the expression of total *GR* (*Nr3c1, GRα + GRβ*) and *Bcat2* mRNAs in quadriceps from P7 animals. While total *GR* mRNA expression is lower in SMA animals, it is not influenced by *Klf15* overexpression (Fig. 7b). Here again, the decreased expression of total *GR* mRNA levels in SMA mice reflects the more abundant *GRa* mRNA, which is also significantly decreased in *Smn*^*−/−*^*;SMN2* and *Smn*^*−/−*^*;SMN2;KLF15 MTg* mice (Fig. 7c), and not that of the *GRβ* mRNA isoform, which is similar between all groups (Fig. 7d). However, *Bcat2* mRNA is similarly increased in both *Smn*^*−/−*^*;SMN2;KLF15 MTg* and *Smn*^*+/−*^*;SMN2;KLF15* MTg mice (Fig. 7e), suggesting that increased KLF15 activity directly impacts BCAA metabolism. We next determined if overexpression of *Klf15* influenced markers of muscle atrophy and pathology in the quadriceps of P7 animals. We observed that the mRNA expression of atrogenes *atrogin-1* and *MurRF-1* was not significantly different between *Smn*^*−/−*^*;SMN2* and *Smn*^*−/−*^*;SMN2;KLF15 MTg* (Fig. 7f, g). Interestingly, overexpression of *Klf15* in control littermates did not increase *atrogin-1* or *MuRF-1* mRNA levels (Fig. 7f, g), which was observed in prednisolone-treated *Smn*^*+/−*^*;SMN2* mice (Fig. 6a, b). The GC-dependent induction of atrophy in control littermates is therefore most likely KLF15-independent. We also did not observe any KLF15-dependent changes in *Myod* and *myogenin* mRNA expression (Fig. 7h, i), suggesting that the difference observed in prednisolone-treated *Smn−/−;SMN2* mice (Fig. 6c, *MyoD* mRNA) and control littermates (Fig. 6d, *myogenin* mRNA) are probably due to KLF15-independent effect of the synthetic GC. Finally, we find a partial restoration of *parvalbumin* mRNA expression in *Smn*^*−/−*^*;SMN2;KLF15 MTg* animals compared to *Smn*^*−/−*^*;SMN2* mice and control littermates (Fig. 7j).

Given that our results highlight potential KLF15-dependent and-independent effects of prednisolone, we next compared weight and survival of untreated and prednisolone-treated *Smn*^*−/−*^*;SMN2*, *Smn*^*+/−*^*;SMN2*, *Smn*^*−/−*^*;SMN2;KLF15 MTg* and *Smn*^*+/−*^*;SMN2;KLF15* MTg mice. We firstly observed that *Smn*^*−/−*^*;SMN2;KLF15 MTg* mice have a significantly greater lifespan than *Smn*^*−/−*^*;SMN2* animals (Fig. 7k), highlighting a KLF15-dependent impact on disease phenotypes*.* Interestingly, prednisolone-treated *Smn*^*−/−*^*;SMN2;KLF15 MTg* mice have a significantly longer lifespan than *Smn*^*−/−*^*;SMN2;KLF15 MTg* mice and *Smn*^*−/−*^*;SMN2* mice treated with prednisolone (Fig. 7k), suggesting a synergistic effect of transgenic *Klf15* overexpression and prednisolone. Comparison of weight curves indeed reflects this, whereby *Smn*^*−/−*^*;SMN2;KLF15 MTg* mice weigh significantly more than *Smn*^*+/−*^*;SMN2* animals during disease progression (Fig. 7l) and prednisolone-treated *Smn*^*−/−*^*;SMN2;KLF15 MTg* mice show an overall increased weight gain compared to both *Smn*^*−/−*^*;SMN2;KLF15 MTg* and prednisolone-treated *Smn*^*−/−*^*;SMN2* animals (Fig. 7l). Finally, weight curves from control littermates show a small but significant weight loss in prednisolone-treated *Smn*^*+/−*^*;SMN2;KLF15* MTg mice compared to *Smn*^*+/−*^*;SMN2* and *Smn*^*+/−*^*;SMN2;KLF15* MTg animals (Fig. 7m). Thus, prednisolone most likely acts via KLF15-dependent and independent mechanisms, potentially in a tissue-specific and systemic manner. These results therefore identify the GC and the KLF15 component of the GC-KLF15-BCAA pathway as separate but interacting therapeutic targets for SMA.

### 3.7 Modulating Downstream GC-KLF15-BCAA Signaling With BCAAs Improves Phenotype in Severe SMA Mice

**Fig. 8:**
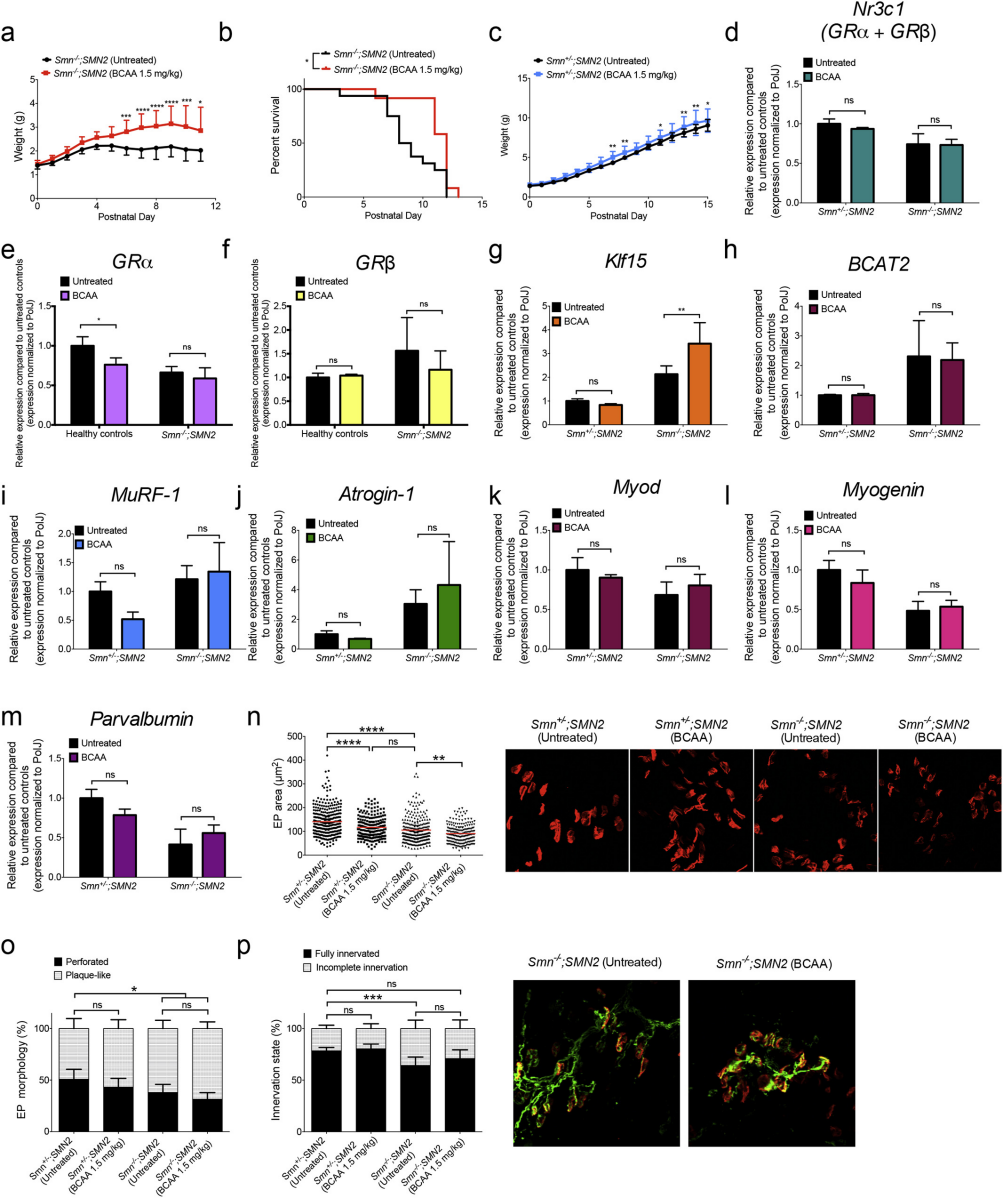
BCAA supplementation improves disease phenotypes of severe SMA mice. *Smn*^*−/−*^*;SMN2* mice and healthy controls were treated with BCAAs (1.5 mg/kg) starting at P5. **a.** Weight curves of BCAA-treated *Smn*^*−/−*^*;SMN2* mice vs. untreated animals. Data represent mean ± SD; *n* = 12–16 animals per group; two-way ANOVA; *p < 0.05, ***p < 0.001, ****p < 0.0001. **b.** Lifespan of BCAA-treated *Smn*^*−/−*^*;SMN2* mice vs. untreated animals. Data represent Kaplan-Meier curves; n = 10–16 animals per group; Log-rank (Mantel-Cox) test; *p* = 0.0159. **c.** Weight curves of BCAA-treated healthy controls vs. untreated animals. Data represent mean ± SD; *n* = 14–18 animals per group; two-way ANOVA; *p < 0.05, **p < 0.01. qPCR analysis of (**d**) *Nr3c1*, (**e**) *GRα*, (**f**) *GRβ*, (**g**) *Klf15*, (**h**) *Bcat2*, (**i**) *MuRF-1*, (**j**) *atrogin-1*, (**k**) *MyoD*, (**l**) *myogenin* and (**m**) p*arvalbumin* mRNAs expression in triceps of BCAA-treated P7 *Smn*^*−/−*^*;SMN2* mice and healthy controls compared to untreated animals. **d-m:** Data represent mean ± SD; n = 3–4 animals per group; two-way ANOVA; **p < 0.01; ns = not significant. **n.** Motor endplate area in TAs of BCAA-treated P7 *Smn*^*−/−*^*;SMN2* mice and healthy littermates compared to untreated animals. Data represent scatter plot ± SD; *n* = 198–324 endplates from 4 animals per group; one-way ANOVA; **p < 0.01, ****p < 0.0001; ns = not significant. **o.** Motor endplate morphology (plaque-like or perforated) in TAs of BCAA-treated P7 *Smn*^*−/−*^*;SMN2* mice and healthy controls compared to untreated animals. Representative images of endplates from untreated and BCAA-treated *Smn*^*−/−*^*;SMN2* mice and healthy littermates. **p.** Innervation status of motor endplates in TAs of BCAA-treated P7 *Smn*^*−/−*^*;SMN2* mice and healthy controls compared to untreated animals. Representative images of NMJs from untreated and BCAA-treated *Smn*^*−/−*^*;SMN2* mice. **o-p**. Data represent mean ± SD; n = 4 animals per group; two-way ANOVA; *p < 0.05, ***p < 0.001; ns = not significant.

Having addressed the functional impact of modulating the upstream signaling cascade of the GC-KLF15-BCAA pathway on SMA pathology, we next wanted to evaluate if modifying downstream activity would display similar benefits. As KLF15 is an activator of BCAA degradation by transcriptional upregulation of *Bcat2,* the first step in BCAA catabolism [4], supplementation of dietary BCAAs may counteract the upregulation of *Klf15* in symptomatic *Smn*^*−/−*^*;SMN2* mice (Fig. 2d, Supplementary Fig. 6). To examine this, *Smn*^*−/−*^*;SMN2* mice and control littermates received daily BCAA supplementation (1.5 mg/kg) by gavage, starting at P5, an early symptomatic time-point. We found that *Smn*^*−/−*^*;SMN2* mice treated with BCAAs display a significant increase in body weight (Fig. 8a) and lifespan (Fig. 8b) compared to untreated mice. Healthy controls also reveal an increased weight gain when treated with BCAAs (Fig. 8c), albeit to a lesser extent.

Similar to our analysis with prednisolone treatment, we assessed the impact of BCAA supplementation on neuromuscular parameters. We first determined the effect of BCAAs on GC-KLF15-BCAA signaling and found that expression of total *GR* mRNA receptor (*Nr3c1, GRα + GRβ*) was unchanged between groups (Fig. 8d). Interestingly, further analysis revealed a small but significant downregulation of *GRα* mRNA levels in BCAA-treated healthy controls (Fig. 8e) while *GRβ* mRNA levels remained similar between groups (Fig. 8f). Whilst *Bcat2* mRNA levels are unchanged between groups (Fig. 8h), *Klf15* mRNA levels are specifically upregulated in muscle from BCAA-treated *Smn*^*−/−*^*;SMN2* mice (Fig. 8g). Finally, BCAA supplementation did not influence *MuRF-1* (Fig. 8i)*, atrogin-1* (Fig. 8j)*, MyoD* (Fig. 8k), *myogenin* (Fig. 8l) and *parvalbumin* (Fig. 8m) mRNA expression in *Smn*^*−/−*^*;SMN2* mice and control littermates.

Interestingly, analysis of endplates reveals a BCAA-induced reduction of area in both healthy controls and *Smn*^*−/−*^*;SMN2* mice compared to untreated animals (Fig. 8n). However, the decreased endplate area did not impact endplate morphology and NMJ innervation as these remained unchanged between BCAA-treated and untreated animals, whereby healthy controls displayed significantly more mature perforated endplates and fully innervated NMJs (Fig. 8o, p). We have previously demonstrated that the size of an endplate does not correlate with its morphology [59]. Combined, our results demonstrate that symptomatic BCAA supplementation leads to significant benefits to a severe SMA mouse model at both a molecular and phenotypic level. While there is an obvious need for a better understanding of the effect of BCAAs on developing muscle and how this may be altered in SMA muscle and other metabolic tissues, we nevertheless provide key evidence that a dietary intervention, implemented at a stage when the neuromuscular decline has begun, can improve disease pathogenesis.

## 4 Discussion

**Fig. 9:**
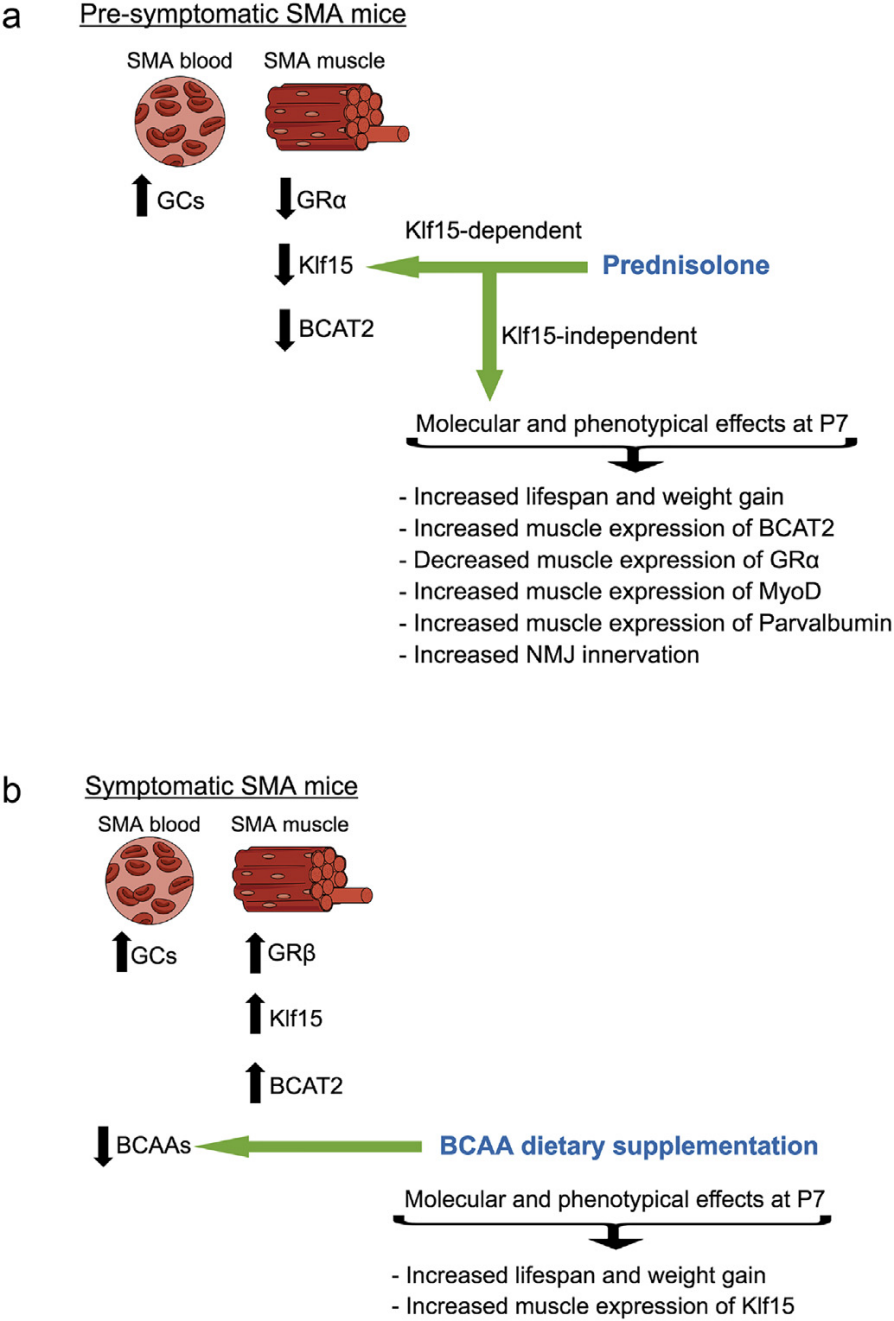
Schematic summarizing the aberrant effectors of the glucocorticoid (GC)-Klf15-branched-chain amino acid (BCAA) signaling cascade targeted by a pre-symptomatic administration of prednisolone (a) and symptomatic BCCA supplementation (b) and the observed effects on molecular, histological and behavioral disease phenotypes.

SMA patients and animal models display diverse metabolic abnormalities [29, 30, 31, 32, 33, 34, 35]. Here, we demonstrate that aberrant expression of the GC-KLF15-BCAA pathway in SMA muscle during disease progression may contribute to muscle and whole-body metabolic perturbations [69]. Indeed, circadian dysregulation of the GC-KLF15-BCAA axis points to intrinsic and systemic metabolic dyshomeostasis. Importantly, through pharmacological and dietary interventions that target GC-KLF15-BCAA signaling, we were able to significantly improve disease phenotypes in 2 distinct SMA mouse models (Fig. 9).

GC activity is mediated via the GR, which is alternatively spliced into two major isoforms: *GRα* and *GRβ* [70]. GRα is thought to be a key mediator of GC-dependent target gene transactivation, while GRβ inhibits GRα and induces GC resistance [46]. Recent studies have also uncovered distinct downstream effectors for GRα and GRβ [71], including a specific role for GRβ in skeletal muscle in the promotion of myogenesis and prevention of atrophy [47]. Our observed increased expression of *GRβ* mRNA in muscle of symptomatic SMA mice (Fig. 1b), may thus be a compensatory attempt to reduce the activity of catabolic pathways (e.g. MuRF-1 and atrogin-1) that accompanies muscle pathology in these mice. In light of SMN's well described housekeeping role in mRNA splicing [72], loss of SMN could have a direct role on the splicing of GR isoforms. However, our analysis of SMA muscle tissue where SMN expression was restored did not reveal an SMN-dependent normalization of *GRα* and *GRβ* mRNA expression (Supplementary Fig. 5b, c). The dysregulated expression of GR isoforms may therefore result from the altered metabolic and pathological status of SMA muscle.

A dysregulated metabolic state may also be responsible for the systemic increased *Klf15* mRNA expression in symptomatic SMA mice. Indeed, *Klf15* activity is directly regulated by GCs, whose key role is to maintain metabolic homeostasis [73]. Several metabolic perturbations have been reported in SMA animal models and patients [74], highlighting an existing metabolically stressed environment that could contribute to the aberrant KLF15 activity in SMA muscle. Seeing as KLF15 is also aberrantly regulated in the muscle disease Duchenne muscular dystrophy (DMD) [55], this transcription factor could act as a key integrator and/or biomarker of the metabolic and pathological state of muscle.

It has previously been demonstrated that increased Klf15 in muscle promotes catabolic pathways by inhibiting the anabolic mTOR signaling [12]. Our observation that the mTOR pathway is significantly downregulated in muscle from symptomatic *Smn*^*−/−*^*;SMN2* mice (Fig. 1f, g) is consistent with this pathological consequence of Klf15 overexpression. This is concurrently accompanied by an increased expression of atrogenes (e.g. Fig.6a, b) [75]. Interestingly, increased mTOR activity may be linked to decreased muscle pathology in milder forms of SMA [76] and loganin-induced benefits in SMA mice are associated with increased mTOR protein synthesis signaling in muscle [77].

Chronic GC administration is known to induce adverse metabolic effects, including wasting of skeletal muscle [78]. An interesting finding throughout this study is the differential effects of GCs, whereby atrophy signaling was induced in healthy animals and ergogenic effects occurred in SMA mice. This dual role of GC administration has previously been reported in DMD *mdx* mice, where GCs had a similar specific benefit on diseased muscle [55]. The absence of phenotypic rescue following the genetic deletion of *atrogin-1* and *MuRF-1* in SMA mice [79] may thus be partly explained by the altered responsiveness of atrophy signaling in SMA muscle. Furthermore, it was recently demonstrated that the dosing regimen itself can influence the balance between catabolic and anabolic effects of GCs in skeletal muscle. Indeed, intermittent dosing of GCs significantly improved skeletal muscle repair and function in mouse models of DMD and Limb-Girdle Muscle Dystrophy while daily administration of GCs promoted muscle atrophy [56,80]. Thus, our dosing regimen of once every two days may also have enhanced the anabolic effects of prednisolone.

In addition, GCs are reported to have gender-specific effects in both adult rodents and humans [81,82]. While SMA is not regarded as a gender-specific disorder, several gender-specific disease modifiers have been reported [83, 84, 85]. Furthermore, there is also evidence to support that certain treatment strategies for SMA have gender-specific outcomes based on the model used [83]. However, in studies where neonatal rodents and horses were exposed to GCs, gender did not influence GC-dependent effects on glucose metabolism (systemic or skeletal muscle), body weight, locomotor activity or motor function (rotarod and grip strength) [86,87]. As we did not discriminate between female and male neonates in our study, we cannot ascertain if prednisolone administration had gender-specific effects in the Taiwanese and *Smn*^*2B/−*^ SMA mice, which would require a more in-depth investigation with larger sample sizes and independent animal models.

To the best of our knowledge, there has never been a clinical trial of GCs in SMA patients. Interestingly, SMA patients in the adeno-associated virus serotype 9 (AAV9)-*SMN1* gene therapy clinical trial also received prednisolone (1 mg/kg) one day pre-gene therapy and for 30 days thereafter [88]. Although prednisolone was used for its immunosuppressive properties, our study suggests that it could have caused additional benefits. There therefore remains a need to better understand the molecular effectors and pathways induced by GCs in healthy, diseased, adult, developing and regenerating muscle of both males and females.

Given the upregulation of *KLF15* across multiple metabolic tissues and spinal cord of SMA mice, BCAA supplementation may have beneficial effects beyond what we observed in skeletal muscle. The decrease of serum BCAA content in symptomatic SMA animals suggests that the metabolic tissues are taking them up in an attempt to compensate for increased *Klf15* activity. BCAAs and aromatic amino acids are precursors of neurotransmitters serotonin and catecholamines, respectively, which compete with each other at the blood-brain barrier to enter the CNS as they use the same transporter [89]. Reduced BCAA levels in SMA serum may increase CNS uptake of aromatic amino acids, directly affecting the synthesis and release of neurotransmitters and overall function.

Prednisolone may also have beneficial effects beyond skeletal muscle, which is highlighted by the observed synergistic benefits of muscle-specific *Klf15* overexpression and systemic prednisolone administration. There is indeed precedence for a role of prednisolone in the pre-synaptic compartment of the NMJ [68], which is reflected in the improved endplate innervation in our prednisolone-treated SMA mice. The anti-inflammatory properties of prednisolone could potentially also modulate aberrant neuroinflammation and immune organ dysfunction recently reported in SMA animals [90, 91, 92]. Nevertheless, our observed striking upregulation of *Klf15* expression in muscle following prednisolone administration suggests a very specific impact on KLF15 signaling. Indeed, the beneficial effect of glucocorticoid treatment in muscle atrophy has long been used in patients suffering from DMD [93]. This ergogenic impact has previously been attributed to the induction of the GC-KLF15 axis [55]. Interestingly, a recent report has identified a synergistic effect of the RhoA/ROCK and GC pathways in muscle of a DMD mouse model [94]. Given that we have previously demonstrated beneficial effects of pharmacological RhoA/ROCK inhibition on survival and neuromuscular phenotype of SMA mice [42,95], the RhoA/ROCK and GC signaling cascades may equally contribute to muscle and metabolic pathologies in SMA muscle. Seeing as therapeutic modulation of the RhoA/ROCK pathway also improves disease phenotypes in neurodegenerative models such as amyotrophic lateral sclerosis and Parkinson's disease [96,97], the perturbed GC-KLF15-BCAA activity may not be limited to SMA and DMD. Thus, investigations on the GC-KLF15-BCAA axis and related therapeutic strategies may have significant repercussions on several neuromuscular and neurodegenerative pathologies.

A surprising observation in our work is that dietary supplementation of BCAAs at a time-point when neurodegenerative and muscle atrophy events have begun is sufficient to significantly improve weight gain and survival in severe SMA mice. Previous studies have shown an influence of diet on SMA disease phenotype but these were fed to the mother or implemented at birth [36, 37, 38]. Interestingly, several SMA patients and their families have adopted an amino acid (AA) diet (http://www.aadietinfo.com/), composed of elemental free form amino acids, including BCAAs. The claimed benefits of the AA diet in SMA patients may thus be reflected in the improved phenotype of SMA mice supplemented with BCAAs and be explained by a perturbed GC-KLF15-BCAA signaling. We are currently planning a small pilot study to investigate BCAA cycling and serum levels of SMA patients and healthy siblings and evaluate how this is influenced by the AA diet. BCAAs have been demonstrated to increase survival and longevity [7,8] as well as promote exercise- and sarcopenia-induced muscle damage repair [9,10]. As such, BCAA supplementation is used by athletes [98] and prescribed for weight regulation [99] and management of sarcopenia [9]. Dysregulated serum levels of BCAAs have also been observed in neurodegenerative diseases such as Huntington's [100], Parkinson's [101] and Alzheimer's [102]. Thus, regulated BCAA supplementation or consumption may have wide-reaching benefits in several neurodegenerative and neuromuscular disorders.

Our work has identified a key role for the GC-KLF15-BCAA axis in SMA pathogenesis, thereby identifying molecular targets to alleviate muscle and metabolic perturbations in SMA. Future therapeutic endeavors should consider a combination of pharmacological and dietary interventions to restore GC-KLF15-BCAA-dependent muscle and metabolic homeostasis alongside SMN-specific treatment strategies [103, 104, 105, 106, 107]. Importantly, the possibility that the GC-KLF15-BCAA pathway may be disrupted in numerous degenerative and metabolic pathologies characterized by muscle loss and wasting combined with the commercial availability of targeted dietary and drug treatment strategies makes it an attractive therapeutic molecular mechanism to further investigate.

## Appendix A Supplementary Data

Supplementary Materials and Methods, Figures, Figure Legends and Table Legend Image 1

Supplementary Table 1 Image 2
